# Diagnostic Yields of Trio-WES Accompanied by CNVseq for Rare Neurodevelopmental Disorders

**DOI:** 10.3389/fgene.2019.00485

**Published:** 2019-05-24

**Authors:** Chao Gao, Xiaona Wang, Shiyue Mei, Dongxiao Li, Jiali Duan, Pei Zhang, Baiyun Chen, Liang Han, Yang Gao, Zhenhua Yang, Bing Li, Xiu-An Yang

**Affiliations:** ^1^Department of Pediatric Rehabilitation Medicine, Children’s Hospital Affiliated to Zhengzhou University/Henan Children’s Hospital/Zhengzhou Children’s Hospital, Zhengzhou, China; ^2^Henan Provincial Key Laboratory of Children’s Genetics and Metabolic Diseases, Children’s Hospital Affiliated to Zhengzhou University/Henan Children’s Hospital/Zhengzhou Children’s Hospital, Zhengzhou, China; ^3^Department of Pediatric Neurology and Rehabilitation, First People’s Hospital of Shangqiu, Shangqiu, China; ^4^Graduate School of Zhengzhou University, Zhengzhou, China; ^5^Third People’s Hospital of Qingdao West Coast New District, Qingdao, China; ^6^Central Laboratory, Jinshan Hospital Affiliated to Fudan University, Shanghai, China; ^7^School of Basic Medical Science, Chengde Medical University, Chengde, China

**Keywords:** neurodevelopmental disorder, whole-exome sequencing, copy number variation sequencing, AIFM1, BRWD3

## Abstract

**Objective:**

This study is to investigate the diagnostic yield of the combination of trio whole exome sequencing (Trio-WES) and copy number variation sequencing (CNVseq) for rare neurodevelopmental disorders (NDDs).

**Methods:**

Clinical data from consecutive pediatric patients who were diagnosed with rare NDDs that were suspected to be monogenic disorders, who were admitted to our hospital from April 2017 to March 2019, and who underwent next generation sequencing (NGS) were extracted from the medical records. Patients for whom Trio-WES and CNVseq data were available were enrolled in this study. Sanger sequencing was applied for the validation of the variants identified by Trio-WES. Sequence alignment and structural modeling were conducted for analyzing the possibility of the variants in the onset of the NDDs.

**Results:**

In total, 54 patients were enrolled in this study, with the median age of 15 (8–26) months. A total of 242 phenotypic abnormalities belonging to 20 different systems were identified in the cohort. Twenty-four patients were diagnosed by Trio-WES, eight patients were diagnosed by CNVseq, and one case was identified by both WES and CNVseq. Compared with Trio-WES, the diagnosis rate of Trio-WES accompanied by CNVseq was significantly higher (*P* = 0.016). Trio-WES identified 36 variants in 26 different genes, among which 27 variants were novel. CNVseq detected four duplications and eight deletions, ranging from 310 kb to 23.27 Mb. Our case examples demonstrated the high heterogeneity of NDDs and showed the challenges of rare NDDs for physicians.

**Conclusion:**

The significantly higher diagnosis rate of Trio-WES accompanied by CNVseq makes this strategy a potential alternative to the most widely used approaches for pediatric children with rare and undiagnosed NDDs.

## Introduction

Neurodevelopmental disorders, which are characterized by delays in global development, motor skills, and cognition, as well as by intellectual disability, affect more than 3% of children (Bellman et al., 2013; Soden et al., 2014). NDDs are rather heterogeneous; therefore, it is difficult to give a clinical diagnosis in some cases. Various detecting methods are involved in the etiologic evaluation of NDDs: biopsies, karyotype, chromosomal microarray analysis (CMA), single gene, and NGS. NGS has emerged as a successful diagnostic tool in the clinic over the past decade, and strategies, including targeted next-generation sequencing panels (Panel), clinical exome sequencing (CES), WES, CNVseq, and whole-genome sequencing (WGS), are extensively used (Xue et al., 2015; Monlong et al., 2018).

The most widely used approaches of NGS are Panel and WES, which have lower cost and more manageable data volumes compared with WGS (Xue et al., 2015). WES has a higher diagnostic yield compared with Panel in monogenic disorders identification; therefore, it is highly recommended (Dillon and Lunke, 2018). CNVs are documented to be risk factors for NDDs, and CMA has been proven as the most stable and accurate platform for the detection of CNV for a decade (Torres et al., 2016; Zhuo et al., 2017). Currently, CNVseq is developed by means of WES or WGS data (Magi et al., 2013; Campbell et al., 2014; Wang et al., 2016). Compared with CMA, CNVseq not only detect mutations found in already known regions but can also provide indicators toward novel disease loci; therefore, it might potentially replace CMA (Martin-Merida et al., 2018; Truty et al., 2018; Wang et al., 2018).

Although a large number of studies regarding NGS, particularly WES, are reported each day, to the best our knowledge, studies about the efficiency of CNVseq in combination with other methods are rarely reported. To this end, we herein investigated the diagnostic yields of Trio-WES accompanied by CNVseq for a cohort of pediatric patients exhibiting NDDs. We intend to provide clinical practice data for strategies selection for pediatric patients referred to genetic counseling.

## Materials and Methods

### Family Recruitment

Patients hospitalized at the Department of Paediatric Rehabilitation Medicine, Zhengzhou Children’s Hospital, between April 2017 and March 2019 showing clinical signs of NDDs but without a diagnosis after clinical and accessory examinations were considered as candidates. These patients were suspected with hereditary disease and had been subjected to NGS. Clinical features of the patients were ascertained by medical record review and physician interviews. The cohort was selected randomly through routine visits but without bias selection to reflect standard clinical practice. The patients, together with their parents, undergoing Trio-WES accompanied by CNVseq were finally included. This study was approved by the Medical Ethics Committee in Children’s Hospital Affiliated to Zhengzhou University, and written informed consent was provided by the parents of the patients. Authorization was obtained from the parents for disclosure of the recognizable persons in photographs.

### Physical and Auxiliary Examination

Upon admission, physical examination especially neurophysical examination and auxiliary examination, were performed to give a clinical diagnosis. Generally, venous blood was collected for routine blood. In addition, laboratory tests for liver function, renal function, procalcitonin, biochemistry, and C-reactive protein were conducted. The following tests were applied to the cohort when needed. Echocardiography was used to detect the pathological changes in lung. Virus and parasite detection including cytomegalovirus-IgG, herpes simplex virus-IgG, rubella virus-IgG, Toxoplasma-IgG, cytomegalovirus-IgM, herpes simplex virus-IgM, rubella virus-IgM, and Toxoplasma gondii-IgM, was undertaken. Blood gas analysis and cerebral spinal fluid examination were performed. Abdominal ultrasonography was used to detect abnormities in abdominal organs. Urinary organic acid analysis was carried out using gas chromatography/mass spectrometry (GC/MS) for the detection of urine metabolite. Video electroencephalogram was applied to monitor patients with epilepsy. Neuromuscular function monitoring was used to detect the state of muscles. Magnetic resonance imaging (MRI) and head computed tomography (CT) scanning were conducted to identify intracranial changes. For some patients, mitochondria DNA sequencing was performed.

### Whole-Exome Sequencing (WES)

Total genomic DNA was extracted with the Blood Genome DNA Extraction Kit DP349 (TianGen, Beijing, China) strictly according to the manufacture’s protocol. A NanoDrop^TM^ spectrophotometer was used to measure DNA purity and concentration. For WES, the method used was as previously described by Tong et al. (2018). The genomic DNA was subjected to sonication (cat: FB705, Thermo Fisher, Waltham, MA, United States) and hybrid capture with xGen Exome Research Panel v1.0 (Integrated DNA Technologies, Inc., Coralville, IA, United States), enriched, and sequenced on the Illumina HiSeq 2000 platform. DNA quality was confirmed by gel electrophoresis during library preparation.

Gene-discovery approach with the use of collaborative phenomics and semiautomated genomics was performed as described previously (Tarailo-Graovac et al., 2016). After raw data were processed by fastp v0.18.1 (Chen et al., 2018), paired-end alignment was performed using the BWA 0.7.8-r455 package to the Ensemble GRCh37/hg19 reference genome (Li and Durbin, 2009, 2010). Base quality score recalibration together with SNP and short Indel calling was conducted with GATK 3.8 (McKenna et al., 2010). According to the sequence depth and mutation quality, SNP and Indel were filtered and screened, and high quality and reliable mutations (mutation depth was at least 2×, mutation rate was more than 10%, mutation quality >20) were obtained using SAMtools 1.6 (Li et al., 2009). Annovar v2017Jul16 (Wang et al., 2010) was employed for variants annotation. Protein biological function prediction were performed based on homology ratio and conservativeness of protein structure with hazard prediction software Provean (Choi and Chan, 2015), Sift, Polypen2_hdiv, Polypen2_hvar (Adzhubei et al., 2010), MutationSotter (Reva et al., 2011), M-Cap, and Revel. MaxEntScan was used to predict shear hazards of mutations near shear sites. The 1,000 genomes project database (1000 Genomes Project Consortium et al., 2010), dbSNP, hapmap, NHLBI exome sequencing project (ESP^1^), and ExAC database^2^ were applied for minor allele frequency (MAF) checking. MAF < 0.5% was used for non-synonymous and LoF exonic variants analysis. Synonymous mutations and intron region mutations were filtered out. The variants identified as the candidate etiologies were classified into “pathogenic,” “likely pathogenic,” “uncertain significance,” “likely benign,” and “benign” according to the American College of Medical Genetics and Genomics and the Association for Molecular Pathology (Richards et al., 2015). Possible pathogenic loci were screened according to three heredity models, namely autosomal recessive (AR) inheritance, autosomal dominant (AD), and X-linked inheritance. Possible pathogenic genes were identified from the screened deleterious variants combining disease correlation and clinical phenotypes.

### CNVseq

For CNVseq, genomic extraction was undertaken with the Blood Genomic DNA Mini Kit using 1 ml peripheral blood strictly according to the protocol provided by the manufacturer (Cwbiotech, Beijing, China). DNA with a total amount of more than 1.2 μg and without RNA contamination was used for further library preparation. Genomic DNA was then broken into 200–300 bp fragments by ultrasound. After library preparation, the samples were subjected to Illumina HiSeq 2500 (Illumina, San Diego, United States). Raw data were processed by the basecall analysis software fastq v0.18.1 (Chen et al., 2018). The clean date were then blasted to human reference genome (hg19) using BWA (Li and Durbin, 2009, 2010). PCR duplications were removed by Picard MarkDuplicates (Ebbert et al., 2016). The mixture-hidden Markov model (m-HMM) approach was used to estimate the window-based copy number change points and copy number states (Wang et al., 2014). An in-house pipeline was applied to map and call CNVs larger than 100 kb. Candidate CNVs were annotated by analyzing the genes contained in the CNVs and the CNV intervals themselves with the databases Decipher, ClinVar, ClinGen, and OMIM. The candidate CNVs were then filtered with normal frequency databases DGV and ISCA. Interpretation of constitutional CNV was undertaken based on annotation information and frequency database according to American College of Medical Genetics standards and guidelines as reported previously (Kearney et al., 2011). The CNVs were divided into five grades as follows: grade 1, aneuploidy reported as definite pathogenicity; grade 2, CNVs reported as likely pathogenic, new CNVs identified by segregation analysis, or genes contained in the CNVs with frequencies less than 0.5% are linked to diseases; grade 3, pathogenic conflicts exist between databases (frequency or possibly pathogenic); grade 4, possible benign in all databases; grade 5, all databases were benign or frequency databases shows a frequency of more than 0.5%.

### Sanger Sequencing

Sanger sequencing was undertaken to validate the candidate pathogenic or likely pathogenic variants identified by Trio-WES. Briefly, amplification of variant containing sequences was performed with an annealing temperature predicted by software DNAclub. The PCR products were then sequenced with ABI 3730XL (Thermo Fisher Scientific Inc., Waltham, MA, United States), followed by analysis with the DNASTAR 5.0 software (DNASTAR, Inc., Madison, WI, United States).

### Statistical Analysis

Statistical analysis was performed using SPSS 23.0 software (SPSS Inc., Chicago, IL, United States). Categorical data were expressed as frequency and percentage, and comparisons between groups were analyzed using chi square. A normality test was first conducted for measurement data. Data that meet normal distribution are expressed as the mean ± SD, and comparisons between groups were conducted using independent sample *t*-test. The data that did not meet a normal distribution are expressed as quartiles, and Mann–Whitney *U* test was used for group comparison. McNemar’s test was used to compare the two diagnostic methods. *P* < 0.05 was considered to indicate a statistically significant difference.

## Results

### Demographics

A total of 54 patients were enrolled in the current study. The detailed information of the demographics of the cohort is shown in Supplementary Table S1. The median age of the patients at diagnosis was 15 (8–26) months; 61.1% (*n* = 33) of the patients were male and male female ratio in this study was 1.57 (33/21). None of the patients were born to consanguineous parents.

### Clinical Characteristics Analysis

**FIGURE 1:**
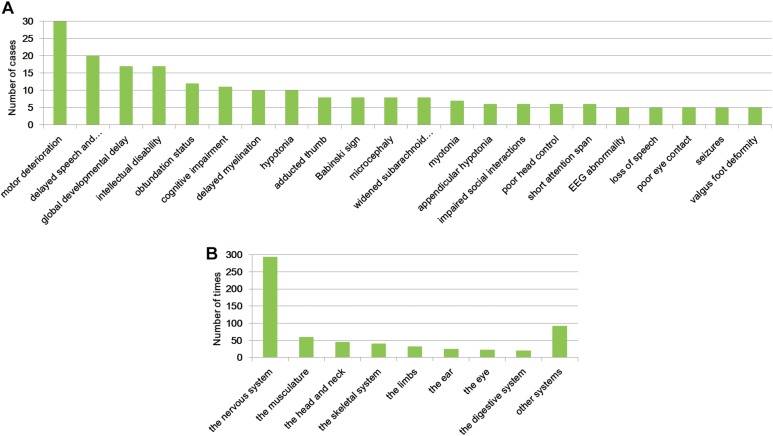
Clinical symptoms analysis. Clinical abnormalities found in more than five patients of the cohort **(A)** and the top eight systems that were affected **(B)**.

To unravel the profile of clinical manifestation for this cohort, clinical characteristics analysis was performed. As shown in Supplementary Table S2, a total of 242 phenotypic abnormalities defined by HPO (The Human Phenotype Ontology^3^) were noted in the patients, with motor deterioration as the most common (35 cases) feature. Twenty-two clinical symptoms were identified in more than five patients (Figure 1A), and eight abnormalities, namely, motor deterioration, delayed speech and language development, global developmental delay, intellectual disability, obtundation status, cognitive impairment, delayed myelination, and hypotonia appeared in no fewer than 10 patients. Except hypotonia, the other features are all related to the nervous system. The identified presentation for the cohort is classified into 20 different systems, with the top eight affected systems (appeared more than 20 times) being the nervous system, the musculature, the head and neck, the skeletal system, the limbs, the ear, the eye, and the digestive system (Figure 1B and Supplementary Table S2).

### Molecular Diagnosis Yields

To provide a molecular diagnosis, Trio-WES accompanied by CNVseq was applied to the cohort and to their parents. Among the patients, 75.9% (41 patients) had undergone trio copy number variation sequencing (Tri-CNVseq). The detailed data quality of WES and CNVseq for the cohort is shown in Supplementary Table S3. After filtering, a molecular diagnosis was made in 57.4% of the cohort (31 cases). For the patients with positive results, 24 patients were diagnosed by WES (Table 1 and Supplementary Table S4), eight patients were diagnosed by CNVseq (Table 2), and one patient was diagnosed by both WES and CNVseq. Chi square and Mann–Whitney *U* test showed no significant differences in sex and age between patients with positive and negative results, respectively (Supplementary Table S5). The variants have been submitted to Clinvar with the accession numbers of SCV000899180–SCV000899223^4^. Additionally, no significant difference in data size, mean depth, coverage, Q20, and Q30 in WES was noted by independent sample *t*-test, indicating that the sequencing operation was stable. Compared with Trio-WES, the McNemar’s test demonstrated that the diagnosis rate of Trio-WES accompanied by CNVseq was significantly higher (*P* = 0.016, Table 3).

**TABLE 1:** WES diagnostic testing characteristics of families with children with developmental delays.

				**Model of**	**Inherited**	
**Patient ID**	**Gene**	**Variants of nucleic acid**	**Variants of protein**	**inheritance**	**from**	**Reported?**
Patient_1	PCDH15	c.5275(exon35)_c.5301(exon35)	p.Pro1759_Pro1767del	AR	Mother	No
		delCCTATTTCTCCTCCTTCTCCTCCTCCT				
Patient_1	PCDH15	c.4337(exon33)_c.4338(exon33)insGCCGCC	p.Pro1446delinsProProPro	AR	Father	No
Patient_2	MED13L	c.6260(exon29)delC	p.Pro2087Glnfs^*^4	AD	*De novo*	No
Patient_3	IGHMBP2	c.1202(exon8)A>G	p.His401Arg	AR	Father	No
Patient_3	IGHMBP2	c.1693(exon12)G>A	p.Asp565Asn	AR	Mother	Yes
Patient_6	ZEB2	c.2177(exon8)_c.2180(exon8)delCTTT	p.Ser726Tyrfs^*^7	AD	*De novo*	No
Patient_8	DHCR7	c.278C>T(exon4)	p.Thr93Met	AR	Father	Yes
Patient_8	DHCR7	c.907G>A(exon8)	p.Gly303 Arg	AR	Mother	Yes
Patient_12	AIFM1	c.452(exon4)G>A	p.Arg151Gln	XR	Mother	No
Patient_13	TCF4	c.1486+2T>G(IVS16)		AD	*De novo*	No
Patient_21	MECP2	gain(EXON:2_4)		XR	Mother	No
Patient_22	BRWD3	c.256(exon5)G>A	p.Glu86Lys	XR	Mother	No
Patient_24	TRIOBP	c.154G>A(exon4)	p.Asp52Asn	AR	Father	No
Patient_24	TRIOBP	c.6548G>A(exon19)	p.Gly2183Asp	AR	Mother	No
Patient_25	NFIX	loss1(EXON:5_6)		AD	*De novo*	No
Patient_32	COL6A1	gain1(EXON:1_35)		AD or AR	*De novo*	No
Patient_34	SMC3	c.1067(exon12)_c.1070(exon12)delAGAA	p.Glu356Glufs^*^46	XD or XR	*De novo*	No
Patient_34	MECP2	c.925(exon4)C>T	p.Arg309Trp	AD	*De novo*	No
Patient_35	OCRL	c.2428(exon22)C>T	p.Arg810Stop,92	XR	*De novo*	No
Patient_36	NDUFS1	c.64(exon3)C>T	p.Arg22Stop,706	AR	Father	No
Patient_36	NDUFS1	c.845(exon9)A>G	p.Asn282Ser	AR	Mother	No
Patient_37	MECP2	c.686(exon4)C>A	p.Ser229Stop,258	XD	*De novo*	No
Patient_38	MLC1	c.206(exon3)C>T	p.Ser69Leu	AR	Father	Yes
Patient_38	MLC1	c.833(exon10)A>G	p.Tyr278Cys	AR	Mother	Yes
Patient_40	GPT2	c.1172(exon9)C>T	p.Pro391Leu	AR	Both	No
Patient_43	NEB	c.11341(exon77)C>T	p.Arg3781Trp	AR	Mother	No
Patient_43	NEB	c.24311(exon171)C>A	p.Ser8104Stop,457	AR	Father	No
Patient_44	PLP1	c.104(exon2)G>A	p.Cys35Tyr	XR	Mother	Yes
Patient_45	PAH	c.728(exon7)G>A	p.Arg243Gln	AR	Father	Yes
Patient_45	PAH	c.875(exon8)C>T	p.Pro292Leu	AR	Mother	Yes
Patient_46	FOXP1	c.1627(exon18)C>T	p.Arg543Stop,134	AD	*De novo*	No
Patient_48	POLR1A	c.2527(exon18)C>T	p.Arg843Stop,878	AD	*De novo*	No
Patient_48	SMN1	c.840C>T(exon7)		AR	Both	No
Patient_50	MMACHC	c.609(exon4)G>A	p.Trp203Stop,80	AR	Both	Yes
Patient_50	DEPDC5	c.1935(exon23)_c.1936(exon23)insA	p.Ser646Lysfs^*^20	AD	*De novo*	No
Patient_53	IFIH1	c.1517(exon7)T>A	p.Ile506Asn	AD	Mother	No

*AR, autosomal recessive; AD, autosomal dominant; XD, X link dominant; XR, X link recessive.*

**TABLE 2:** Rare CNVs identified by CNVseq.

				**Genes linked to**		
				**diseases/genes**		
**Patient ID**	**Position (hg19)**	**Size (bp)**	**Inheritance**	**in this region**	**Genes included**	**Prediction**
Patient_7	Dup 175843728-180703728, 5q35.2-q35.3	4.86 Mb	*De novo*	21/77	***SNCB, NSD1, SLC34A1, F12, DDX41, B4GALT7, PROP1, NHP2, AGXT2L2, GRM6, ADAMTS2, LTC4S, SQSTM1, FLT4*** FAF2, RNF44, CDHR2, GPRIN1, EIF4E1B, TSPAN17, UNC5A, HK3, UIMC1, ZNF346, FGFR4, RAB24, PRELID1, MXD3, LMAN2, RGS14, PFN3, GRK6, PRR7, DBN1, PDLIM7, DOK3, FAM193B, TMED9, FAM153A, N4BP3, RMND5B, HNRNPAB, COL23A1, CLK4, ZNF354A, ZNF354B, ZFP2, ZNF454, ZNF879, ZNF354C, RUFY1, HNRNPH1, C5orf60, CBY3, CANX, MAML1, MGAT4B, C5orf45, TBC1D9B, RNF130, RASGEF1C, MAPK9, GFPT2, CNOT6, SCGB3A1, OR2Y1, MGAT1, ZFP62, BTNL8, BTNL3, BTNL9, OR2V1, OR2V2, TRIM7, TRIM41, GNB2L1, TRIM52	Probably pathogenic
	Del 162966301-170914973, 6q26-q27	7.95 Mb	*De novo*	15/35	***PARK2, PDE10A, T, MPC1, RNASET2, SMOC2, THBS2, C6orf70, TBP*** PACRG, QKI, C6orf118, PRR18, SFT2D1, RPS6KA2, FGFR1OP, CCR6, GPR31, TCP10L2, UNC93A, TTLL2, TCP10, MLLT4, HGC6.3, KIF25, FRMD1, DACT2, WDR27, C6orf120, PHF10, TCTE3, DLL1, FAM120B, PSMB1, PDCD2	Probably pathogenic
Patient_9	Dup 12546855-35816855, 8p23.1-p12	23.27 Mb	*De novo*	27/125	SLC39A14, LPL, LZTS1, GTF2E2, GSR, FGF20, SFTPC, PDGFRL, DLC1, TUSC3, TTI2, VPS37A, ASAS1, HR, BMP1, NAT2, NEFL, NKX2-6, EPHX2, TNFRSF10B, CHRNA2, MSR1, EXTL3, FGF17, GNRH1, ATP6V1B2, WRN ESCO2, ADAM28, ADAM7, ADAMDEC1, ADRA1A, BIN3, BNIP3L, C8orf48, C8orf58, CCDC25, CDCA2, CHMP7, CLU, CNOT7, CSGALNACT1, DCTN6, DOCK5, DOK2, DPYSL2, DUSP26, DUSP4, EBF2, EFHA2, EGR3, ELP3, ENTPD4, EPB49, FAM160B2, FBXO16, FGL1, FUT10, FZD3, GFRA2, HMBOX1, INTS10, INTS9, KCTD9, KIAA1456, KIAA1967, KIF13B, LEPROTL1, LGI3, LOC100507341, LOC101059966, LONRF1, LOXL2, MAK16, MBOAT4, MTMR7, MTUS1, NAT1, NEFM, NKX3-1, NPM2, NRG1, NUDT18, NUGGC, PBK, PCM1, PDLIM2, PEBP4, PHYHIP, PIWIL2, PNMA2, PNOC, POLR3D, PPP2CB, PPP2R2A, PPP3CC, PSD3, PTK2B, PURG, R3HCC1, RBPMS, REEP4, RHOBTB2, RNF122, SCARA3, SCARA5, SGCZ, SH2D4A, SLC18A1, SLC25A37, SLC7A2, SORBS3, STC1, STMN4, TEX15, TMEM66, TNFRSF10A, TNFRSF10C, TNFRSF10D, TRIM35, UBXN8, UNC5D, XPO7, ZDHHC2, ZNF395	Probably pathogenic
	Del 155001-6955001, 8p23.3-p23.1	6.80 Mb	*De novo*	3/22	***CLN8, ARHGEF10, MCPH1*** AGPAT5, ANGPT2, C8orf42, CSMD1, DEFA1, DEFA1B, DEFA3, DEFA4, DEFA5, DEFA6, DEFB1, DLGAP2, ERICH1, FBXO25, KBTBD11, LOC100996633, MYOM2, XKR5, ZNF596	Probably pathogenic
Patient_10	Del 58024137-77996821, 18q21.32-q23	19.97 Mb	n/a	12/60	***BCL2, CNDP1, CTDP1, CYB5A, MC4R, PIGN, RTTN, SERPINB7, SERPINB8, TNFRSF11A, TSHZ1, TXNL4A*** ADNP2, ATP9B, C18orf62, C18orf63, CBLN2, CCDC102B, CD226, CDH19, CDH20, CDH7, CNDP2, DOK6, DSEL, FAM69C, FBXO15, GALR1, HMSD, HSBP1L1, KCNG2, KDSR, KIAA1468, LOC100996274, MBP, NETO1, NFATC1, PARD6G, PHLPP1, PQLC1, RBFA, RNF152, SALL3, SERPINB10, SERPINB11, SERPINB12, SERPINB13, SERPINB2, SERPINB3, SERPINB4, SERPINB5, SOCS6, TIMM21, TMX3, VPS4B, ZADH2, ZCCHC2, ZNF236, ZNF407, ZNF516	Pathogenic
Patient_21	Dup 15323210-153542100, Xq28-q28	310 Kb	n/a	4/9	***HCFC1, MECP2, OPN1LW, OPN1MW*** IRAK1, OPN1MW2, TEX28, TKTL1, TMEM187	Pathogenic
Patient_26	Del 22751194-23251194, 15q11.2-q11.2	0.5 Mb	n/a	4/4	***CYFIP1, NIPA1, NIPA2, TUBGCP5***	Probably pathogenic
Patient_27	Del 162485583-168295583, 2q24.2-q24.3	5.81 Mb	*De novo*	6/20	***IFIH1, SCN2A, GALNT3, TTC21B, SCN1A, SCN9A*** SLC4A10, DPP4, GCG, FAP, GCA, KCNH7, FIGN, GRB14, COBLL1, SLC38A11, SCN3A, CSRNP3, SCN7A, XIRP2	Pathogenic
Patient_32	Dup43010560-48093051, 21q22.3-q22.3	5.08 Mb	*De novo*	18/79	***RIPK4, TMPRSS3, RSPH1, NDUFV3CBS, CRYAA, SIK1, CSTB, AIRE, TSPEAR, ITGB2, COL18A1, COL6A1, COL6A2, FTCD, LSS, PCNT*** CELSR1, GRAMD4, CERK, TBC1D22A, FAM19A5, BRD1, ZBED4, CRELD2, PIM3, IL17REL, TTLL8, MOV10L1, PANX2, TRABD, SELO, HDAC10, MAPK12, MAPK11, PLXNB2, DENND6B, PPP6R2, ADM2, MIOX, LMF2, NCAPH2, ODF3B, KLHDC7B, SYCE3, CPT1B, MAPK8IP2	Probably pathogenic
	Del46794432-51139778, 22q13.31-q13.33	4.35 Mb	*De novo*	11/39	***ALG12, MLC1, TUBGCP6, SBF1, SCO2, TYMP, CHKB, ARSA, SHANK3*** CELSR1, GRAMD4, CERK, TBC1D22A, FAM19A5, BRD1, ZBED4, CRELD2, PIM3, IL17REL, TTLL8, MOV10L1, PANX2, TRABD, SELO, HDAC10, MAPK12, MAPK11, PLXNB2, DENND6B, PPP6R2, ADM2, MIOX, LMF2, NCAPH2, ODF3B, KLHDC7B, SYCE3, CPT1B, MAPK8IP2	Probably pathogenic
Patient_49	Del72682338-74141250, 7q11.23-q11.23	1.46 Mb	*De novo*	10/26	***ELN, STX1A, BAZ1B, CLIP2, GTF2I, GTF2IRD1, LIMK1, RFC2, TBL2, MLXIPL*** ABHD11, BCL7B, CLDN3, CLDN4, DNAJC30, EIF4H, FKBP6, FZD9, LAT2, LOC100996451, NSUN5, TRIM50, VPS37D, WBSCR22, WBSCR27, WBSCR28	Probably pathogenic

*Del, deletion; Dup, duplication; n/a, not available. Bolded terms: genes reported to be associated with diseases.*

**TABLE 3:** A combination of WES and CNVseq was significantly effective than WES in patients with rare neurological disorders.

		**WES + CNVseq**		***P***
		**Negative**	**Positive**	
WES	Negative	23	7	0.016
	Positive	0	24	

### Variant Characteristics

For the 24 patients diagnosed by WES, autosomal dominant disorder (AD), autosomal recessive disease (AR), X-linked dominant disorder (XD), and X-linked recessive disease (XR) were diagnosed in 10, 20, 2, and 6 cases, respectively, and three patients were found to have two mutant genes (Table 1). Thirty-six variants were identified in 26 different genes, among which, 27 variants were novel (75.0%). Additionally, 16 (44.4%) variants were compound heterozygous inherited from the parents, 12 (33.3%) variants were identified to be *de novo*, five (13.9) arose as a result of maternal mutation, and three (8.3%) were homozygous mutation inherited from the parents. CNVseq identified 11 pathogenic or probably pathogenic CNVs in eight patients (five cases underwent Trio-CNVseq). Four CNVs were duplications and seven were deletions, ranging from 310 kb to 23.27 Mb (Table 2 and Supplementary Figure S1). These 11 variants were located in 10 chromosomes, among which, two CNVs in the same chromosome were demonstrated in patient_9. Of note, for the patients with a negative result, uncertain results were found in two (5.1%) patients.

### Case Examples

#### Patient_12

Patient_12 was a 3-month-10-day-old male infant admitted to our hospital due to “discontinuous seizures for more than 3 months.” The boy was born at 38 + 2 weeks by normal vaginal delivery after an uneventful pregnancy and perinatal period to a G1P1 female, with a birth weight of 3.2 kg. Neonatal jaundice was noted and cured by blue light. A convulsive seizure occurred 3 days after birth, and the boy twitched three to four times each day without fever. He was sent to a local hospital and treated with ganglioside and oral vitamin AD drops for more than 1 month. When he was 2 months old, seizures without apparent cause occurred again, starting from two times a day and gradually to seven to eight times each day, accompanied by painful crying but without fever, vomiting, diarrhea, etc. He then visited our hospital and was diagnosed with “epileptic spasm.” Seizures were basically relieved after he was given phenobarbitone tablets, Keppra, and oral sodium valproate.

**FIGURE 2:**
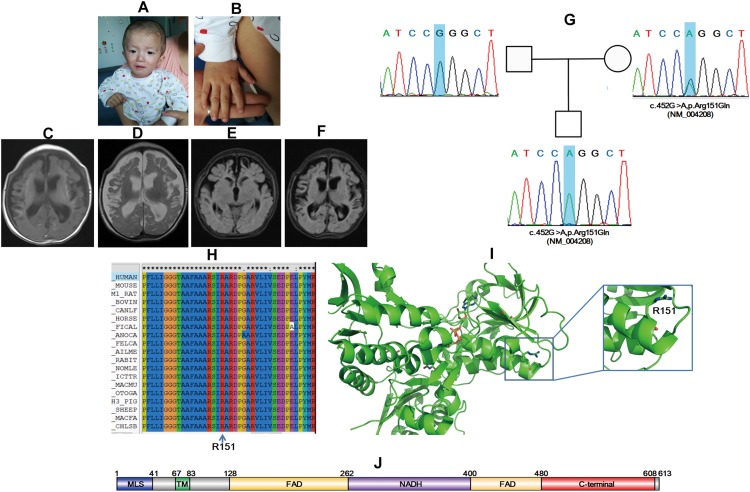
*AIFM1* variant induces X-linked COXPD6 in a 3-month-10-day-old male infant. Premature anterior fontanelle closure **(A)**, hand clenching **(A)**, and adducted thumb were noted **(B)**. Softened left frontal lobe and dilation of lateral ventricles were found **(C–F)**. Sanger sequencing showed that the proband had a novel mutation inherited form his mother **(G)**. Amino sequence alignment indicated that the mutation site is conserved among different species **(H)** and structural modeling revealed that R151 locate on an α helix, and the R151E variant might be pathogenic **(I)**. Schematic map of protein domains for AIFM1 **(J)**.

A scattered red pinpoint-size skin rash was found around the face, which retreated after pressing. Premature anterior fontanelle closure was noted, and cranial suture overlap of the frontal and parietal occipital lobes was obvious (Figure 2A). Hand clenching and an adducted thumb were noted (Figures 2A,B). A response of the auditory and visual test was not elicited. The infant had myotonia, and neuromuscular function monitoring revealed increased tension on both sides of the gastrocnemius muscle and hamstring during passive drafting. Congenital metabolic deficiency urine screening demonstrated lactic acid urine accompanied with glycerol and dicarboxylic acid increase and VPA+. Reproductive-related infectious disease tests showed that the patient was cytomegalovirus-IgG-positive and cytomegalovirus-IgM-positive and cytomegalovirus DNA detection result was <5.00E+02 copies/ml. An electrocardiogram indicated sinus tachycardia. A head MRI showed bilateral frontal temporal parietal lobe atrophy and peripheral cortical necrosis. Softened left frontal lobe and dilation of lateral ventricles were found (Figures 2C–F).

Trio-WES together with Trio-CNVseq was applied to the family. Trio-CNVseq was negative, whereas Trio-WES showed that the proband was with a hemizygous variant of c.452G>A,p.R151Q (NM_004208) in *AIFM1*. *AIFM1* is documented to be responsible for the onset of combined oxidative phosphorylation deficiency 6 (COXPD6, MIM: 300816). The identified variant is recorded as rs752742151, and in addition to ExAC (MAF 0.0001), it is not included in any database. Standard interpretation according to ACMG guidelines (Richards et al., 2015) showed that the variant was PM1 + PM2 + PM and met the standard of “likely pathogenic.” The father matched the reference sequence, and the mother was heterozygous for this site, which was validated by Sanger sequencing (Figure 2G). Sequence alignment showed that the whole AIFM1 protein including R151, is highly conserved among species. Structural modeling indicated that R151 is located at the C terminal of the first α helix of 4lii (Figures 2H,I). The N terminal TA together with the liner loop region GGG, are involved in binding to the substrate, FADA. The R151Q mutation might disrupt the local structure of the first α helix, which in turn influences the binding of AIFM1 to FADA. In line with the aforementioned results, *AIFM1* was considered as etiology, and the proband was diagnosed as COXPD6.

#### Patient_22

Patient_22 was a boy admitted to our hospital due to “difficulty standing” when he was 1 year and 8 months old. The mother was G2P2, and the boy had a healthy 4-year-old elder sister. Fetus preservation was undertaken at the initial stage of pregnancy due to bloody show. He was born at 32 weeks by normal vaginal delivery, with a birth weight of 3.3 kg. The proband could sit and turn over at 8 months and could speak at 1 year and 6 months; however, he could not stand alone. He preferred to shake his head during talking. No family member with such features existed.

**FIGURE 3:**
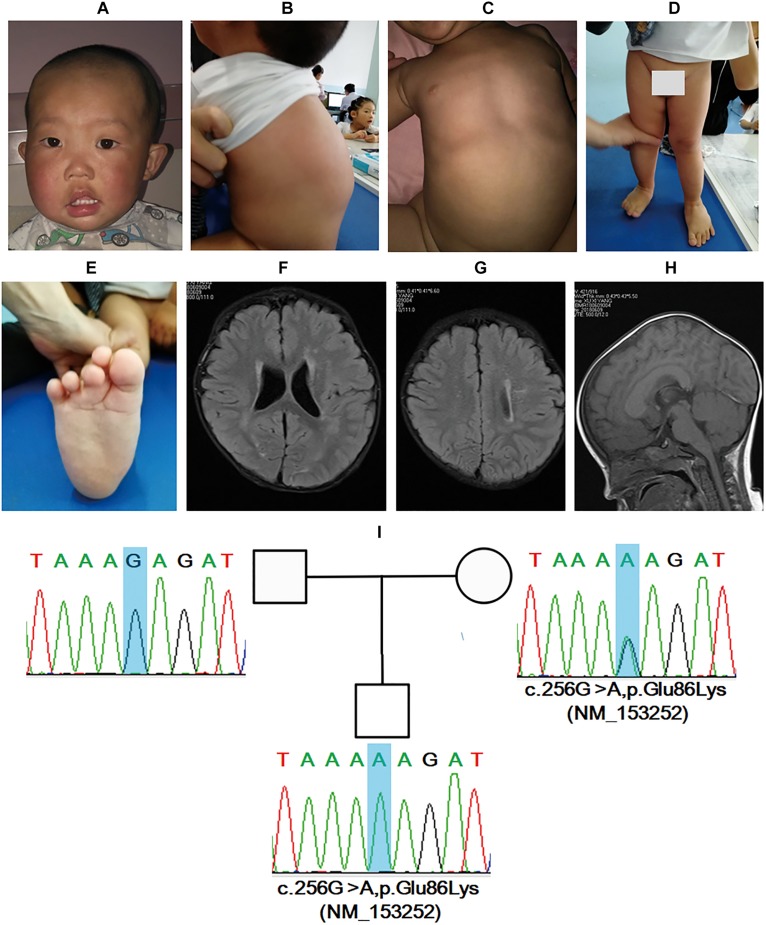
*BRWD3* variant induces mental retardation, X-linked 93 in a 1-year-and-8-month-old male patient. Facial dysmorphic features, including asymmetry, high forehead, hypertelorism, downturned corners of mouth, strabismus, drooling, and protruding ears were noted **(A)**. Multiple bulging masses in the back, slight pectus excavatum, lower limb asymmetry, and pes planus were revealed **(B–E)**. Head MRI showed abnormalities in bilateral frontal and parietal white matter together with corpus callosum **(F–H)**. Sanger sequencing demonstrated that the boy had a novel mutation inherited form his mother **(I)**.

Skull asymmetry was revealed and the head circumference was 48 cm. Delays in intelligence, motor, and language development, hemifacial hypertrophy (right), facial asymmetry, high forehead, hypertelorism, downturned corners of the mouth, strabismus, drooling, and protruding ears were found (Figure 3A). Facial ultrasound showed thickening of the subcutaneous fat layer and abnormality of the subcutaneous and muscular tissue. Additionally, multiple bulging masses in the back were noted (Figure 3B), and ultrasound demonstrated subcutaneous fat layer thickening. Slight pectus excavatum and lower limb asymmetry were revealed, and the right leg was approximately 0.5 cm larger than the left leg (Figures 3C,D). The boy had pes planus, and the second toe was crossed with the third toe in the right foot (Figure 3E). A head MRI showed multiple punctiform and patchy high signals on FLAIR sequences and demyelination in bilateral frontal and parietal white matter (Figures 3F,G). In addition, a thick corpus callosum was identified (Figure 3H).

Trio-WES demonstrated a hemizygous variant of c.256G>A,p.Glu86Lys (NM_153252) in *BRWD3*. *BRWD3* mutations are identified to be the etiology of mental retardation, X-linked 93 (OMIM:300659). The variant for the patient is not included by any database to date. According to ACMG guideline (Richards et al., 2015) interpretation, the variant was PM1 + PM2 + PM1 and met the standard of “likely pathogenic.” The variant was inherited from his mother and was validated by Sanger sequencing (Figure 3I). Collectively, the boy was diagnosed with mental retardation, X-linked 93.

## Discussion

In this study, Trio-WES accompanied by CNVseq was undertaken for 54 pediatric patients with rare NDDs, and high diagnosis yields (57.4%) were achieved. We studied the clinical abnormalities of the cohort and found that the musculature, the head and neck, the skeletal system, the limbs, the ear, the eye, and the digestive system are highly affected, in addition to the nerve system. We also found that Trio-WES accompanied by CNVseq could significantly improve the diagnosis rate for NDDs compare with that of Trio-WES, and our results have broad implications for pediatric medicine.

We herein studied the clinical features for all participates to give an overall profile of the cohort and provide practical data for clinical practice in the future. In total, 242 phenotypic abnormalities encountered in these patients were identified. The most noted features found in the cohort were motor deterioration, delayed speech and language development, global developmental delay, intellectual disability, obtundation status, cognitive impairment, delayed myelination, and hypotonia. These performances are highly consistent with the definition of NDDs, which affects the four domains of gross and fine motor skills, language, social and personal activities, and cognition (Bellman et al., 2013; Soden et al., 2014). The 242 phenotypic abnormalities are related 20 different systems, with the nervous system as the most commonly implicated. In addition, the musculature, the head and neck, the skeletal system, the limbs, the ear, the eye, and the digestive system were also highly affected. We suggest that for the diagnosis of patients with NDDs, abnormality of these systems should be given full attention.

In this study, Trio-WES accompanied by CNVseq was conducted for the cohort to provide a molecular diagnosis for the cohort, and finally, a high diagnostic yield of 57.4% (31/54) was acquired. No difference was found in gender, age, and five key sequencing indicators between patients with positive and negative results, thereby, we consider that the results are not influenced by operation. For Trio-WES, the diagnosis rate was 44.4% (24/54), which, is similar to that of Dillon and Lunke (2018) (42%) and our previous study (38.7%, 12/31) (Tong et al., 2018). It is worth noting that some patients were with uncertain results, therefore, a higher rate might be got with the deepening of our understanding of rare diseases. CNVseq was previously documented to be contribute to 7.1–11% of the molecular diagnosis solved rate (Gambin et al., 2017), and the positive rate for CNVseq in this study was 14.8% (8/54). Trio-WES accompanied by CNVseq was more powerful than Trio-WES. In line with this, we propose that the strategy of Trio-WES accompanied by CNVseq should be widely applied for pediatric patients with rare and undiagnosed NDDs.

We have identified 36 pathogenic or likely pathogenic variants in 26 genes. For the variants, *de novo* mutations were found in a high proportion of the cohort, and this is consistent with previous studies (Franz et al., 2013; Soden et al., 2014). CNVs were identified in eight patients, and four patients had pathogenic variants, while the other four had probably pathogenic variants. The possibility of pathogenicity for probably pathogenic variants was based on functional analysis of the genes enrolled. Although our result for the identification of etiology of the probable pathogenic variants combined multiple lines of evidence, further functional studies are needed to validate our conclusion. In this study, 75.9% (41/54) of the patients had undergone Trio-CNVseq. It should be noted that the proband only model might cause a false-positive result. For example, a 310 kb CNV in 15q13.3 (Dup32024444-32334444,15q13.3-q13.3) was first identified in patient_19; however, that variant was found to be inherited from the father after the parents were subjected to CNVseq. As a result, we consider that Trio-CNVseq should be applied to reduce the false-positive rate.

The male female ratio in this study was 1.57 (33/21), which was higher than the demographic structure of China (approximately 1.15). The reason might be that males are susceptible to X-chromosome-related hereditary diseases. As the sample size was small, whether gender could affect the morbidity of rare NDDs needs further large sample investigation. Among the genes identified in the cohort, X-linked genetic diseases accounted for 26.9% (7/26). In these seven patients, six were found to have mental retardation, while the other case was too young to draw a conclusion. In line with these results, physicians should pay special attention to X-linked mental retardation (XLMR) as it is a notable model of genetic heterogeneity in patients (Tarpey et al., 2009).

Patient_12 in this study highlighted the challenges for disease-causing variant identification even with large-scale sequencing screens. A novel hemizygous mutation of c.452G>A,p.R151Q (NM_004208) in *AIFM1* was found to be the molecular etiology. *AIFM1* is a gene of 36.471 kb in length that is located on Xq25-q26. *AIFM1* contains 16 exons and encodes a protein of 613 amino acids (Susin et al., 1999). AIF is localized in mitochondria, and when an apoptotic injury occurs, it is transported from mitochondria to the nucleus, hence causing programmed cell death (Joza et al., 2001). In addition to the function as a caspase-independent apoptotic effector, AIF plays important roles in hearing and genetic metabolism and development and could lead to relative abnormalities, such as auditory neuropathy spectrum disorder (ANSD), mitochondrial encephalopathy, and Cowchock syndrome (Ghezzi et al., 2010; Rinaldi et al., 2012; Zong et al., 2015). Three features have been classified in Online Mendelian Inheritance in Man, namely, COXPD6, Deafness, X-linked 5 (DFNX5, MIM: 300614), and Cowchock syndrome (COWCK, MIM: 310490). Additionally, AIF mutations are responsible for diseases such as early prenatal ventriculomegaly and infantile motor neuron disease (Berger et al., 2011; Diodato et al., 2016), making diagnosis more complicated. We intended to investigate whether these deferent clinical phenotypes were caused by mutations at different domains to help diagnosis according to the mutated sites; however, we could not draw such a conclusion. For example, mutations at NADH, FAD, and the C terminal domains (Figure 2J) could all bring about the occurrence of DFNX5 (Zong et al., 2015).

*BRWD3* is documented to be associated with the onset of mental retardation, X-linked 93, and the identified variants were truncated mutation or partial deletions (Field et al., 2007; Tarpey et al., 2009; Grotto et al., 2014). We herein reported a boy with a missense mutation in *BRWD3*. To the best of our knowledge, this is the first report of a patient with XLMR caused by a missense mutation in *BRWD3*. Grotto et al. (2014) have summarized the clinical spectrum of BRWD3-related intellectual disability. Most of the patients had mild to moderate intellectual disability, speech delay, dysmorphic facial features, and skeletal symptoms. The clinical features of the proband in this study overlapped with most of the characteristics of the patients reported; therefore, the molecular diagnosis was reasonable. Subcutaneous fat layer thickening and abnormality of subcutaneous and muscular tissue in the face and back were noted in the boy in the present study, which was not reported previously. In line with this, this feature might not be caused by the mutation in *BRWD3.* However, analysis using The Human Protein Atlas^5^ showed that BRWD3 is highly expressed in skin; therefore, the association between this abnormality and mental retardation, X-linked 93 remains to be further elucidated. A head MRI demonstrated a thick corpus callosum and high signals in bilateral frontal and parietal white matter in the patient, which was not reported previously. The head MRI for Patient_1 from Family 15709 of Grotto et al. (2014) at the age of 16 was normal. As a result, whether head MRI abnormalities are classical features of mental retardation, X-linked 93 remain to the further studied. However, as cerebral change is common in patients with mental retardation, it is possible to assume that this feature is caused by the mutation.

Collectively, we herein investigated the diagnosis yields of Trio-WES accompanied by CNVseq in pediatric patients with rare NDDs. To the best of our knowledge, no similar study has been performed previously; therefore, our study provided piratical clinical data for the formulation of diagnostic strategies. It should be noted that the sample size in this study was small and further studies with a larger sample size are needed to provide more reliable data. By studying the profile of the cohort, the head and neck, the ear, and the eyes were found to be most affected in addition to the nerve system; therefore, a pediatrician should pay more attention to these systems during a consultation.

## Ethics Statement

This study was approved by the Medical Ethics Committee in Children’s Hospital Affiliated to Zhengzhou University, and written informed consent was provided by the parents of the patients. Authorization has been obtained from the parents for disclosure of the recognizable persons in photographs.

## Author Contributions

CG, XW, SM, DL, JD, PZ, BC, LH, and YG collected the clinical data. ZY performed the statistical analysis. CG and BL designed the study. X-AY conceived and designed the study, performed data analysis, and wrote the manuscript. All authors contributed to data acquisition and data interpretation, critically revised the manuscript for important intellectual content, approved the final, submitted version of the manuscript, and agreed to be accountable for all aspects of the work.

## Conflict of Interest Statement

The authors declare that the research was conducted in the absence of any commercial or financial relationships that could be construed as a potential conflict of interest.

## Footnotes

1 http://evs.gs.washington.edu/EVS/

2 http://exac.broadinstitute.org

3 http://human-phenotype-ontology.github.io/

4 https://www.ncbi.nlm.nih.gov/clinvar/

5 https://www.proteinatlas.org/ENSG00000165288-BRWD3/tissue

## Supplementary Material

FIGURE S1 Ten copy number variants were identified in seven patients by means of CNVseq. Patient_7, Dup 175843728-180703728, 5q35.2-q35.3 (up panel) Del 162966301-170914973, 6q26-q27 (lower panel) **(A)**; Patient_9, Dup 12546855-35816855, 8p23.1-p12 (up panel), Del 155001-6955001, 8p23.3-p23.1 (lower panel) **(B)**; Patient_10, Del 58024137-77996821,q21.32-q23 **(C)**; Patient_21, Dup 15323210-153542100, Xq28-q28 **(D)**; Patient_26, Del22751194-23251194,15q11.2-q11.2 **(E)**; Patient_27, Del162485583-168295583, 2q24.2-q24.3 **(F)**; Patient_32, Dup43010560-48093051,21q22.3-q22.3 (up panel), Del46794432-5113977822q13.31-q13.33 (lower panel) **(G)**. Patient_49, Del72682338-74141250, 7q11.23-q11.23 **(H)**. Proband, father, mother for **(A,B,E,G,H)**.

TABLE S1 Demographics and clinical feature HPO input and output of the cohort.

TABLE S2 Frequency of the systems affected in the cohort.

TABLE S3 The detailed data quality of WES and CNVseq for the cohort.

TABLE S4 Detailed information of the variants identified in the cohort by Trio-WES.

TABLE S5 Comparison of general clinical information and sequencing data in patients with a positive and negative molecular diagnosis.

## Abbreviations

CNVseq: copy number variation sequencing
NDD: neurodevelopmental disorder
NGS: next generation sequencing
Trio-WES: trio whole-exome sequencing
WES: whole-exome sequencing.
