# Supplementary figures and images for: Diagnostic Yields of Trio-WES Accompanied by CNVseq for Rare Neurodevelopmental Disorders
Source: Front Genet. 2019 May 24;10:485. doi: 10.3389/fgene.2019.00485 (PMC6542989; doi:10.3389/fgene.2019.00485)

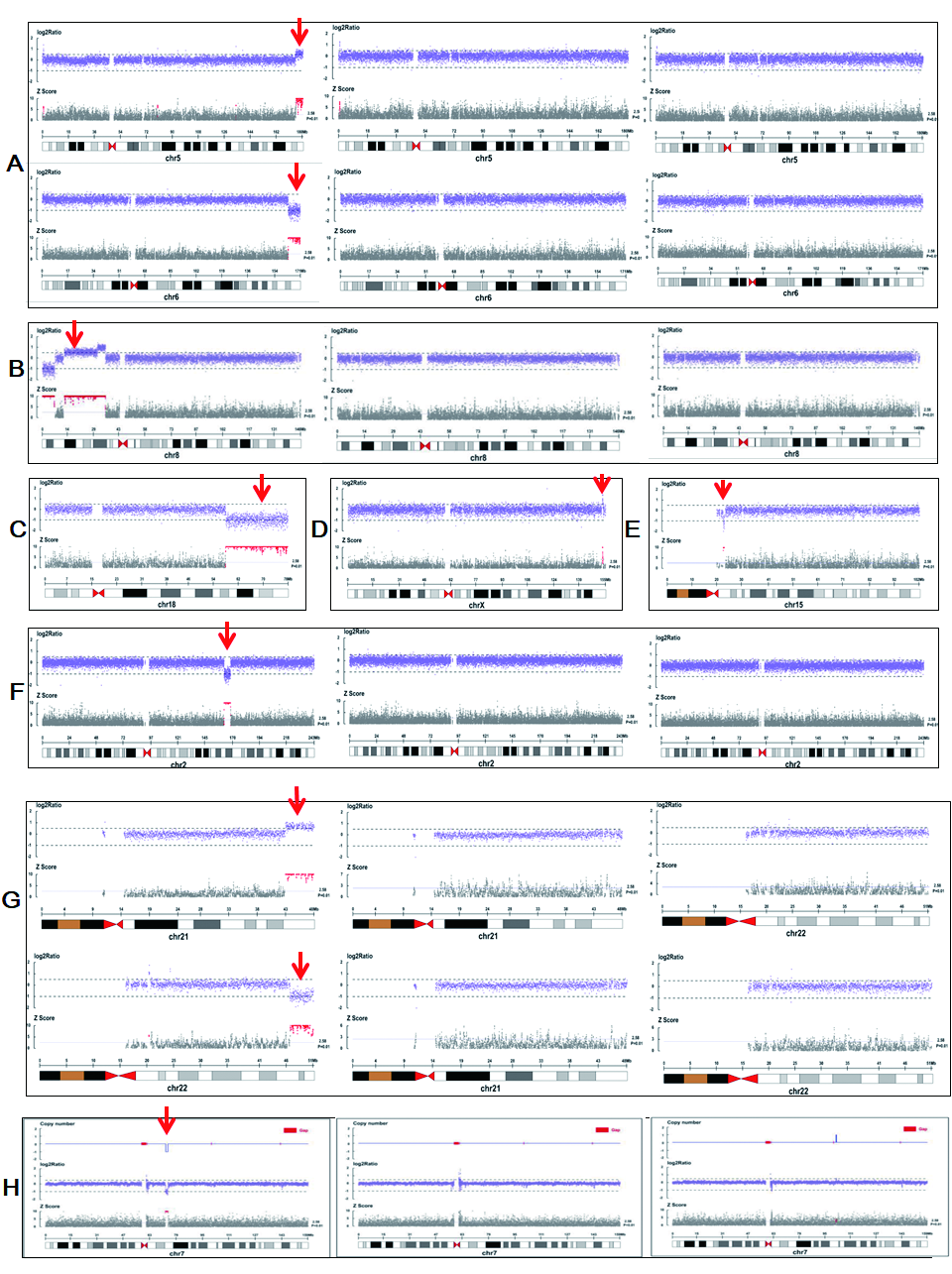

Supplement: FIGURE S1 — Ten copy number variants were identified in seven patients by means of CNVseq. Patient_7, Dup 175843728-180703728, 5q35.2-q35.3 (up panel) Del 162966301-170914973, 6q26-q27 (lower panel) (A); Patient_9, Dup 12546855-35816855, 8p23.1-p12 (up panel), Del 155001-6955001, 8p23.3-p23.1 (lower panel) (B); Patient_10, Del 58024137-77996821,q21.32-q23 (C); Patient_21, Dup 15323210-153542100, Xq28-q28 (D); Patient_26, Del22751194-23251194,15q11.2-q11.2 (E); Patient_27, Del162485583-168295583, 2q24.2-q24.3 (F); Patient_32, Dup43010560-48093051,21q22.3-q22.3 (up panel), Del46794432-5113977822q13.31-q13.33 (lower panel) (G). Patient_49, Del72682338-74141250, 7q11.23-q11.23 (H). Proband, father, mother for (A,B,E,G,H). [file Image_1.tif]
