# Supplementary material for: Diagnostic Yields of Trio-WES Accompanied by CNVseq for Rare Neurodevelopmental Disorders
Source: Front Genet. 2019 May 24;10:485. doi: 10.3389/fgene.2019.00485 (PMC6542989; doi:10.3389/fgene.2019.00485)
Supplement: TABLE S5 — Comparison of general clinical information and sequencing data in patients with a positive and negative molecular diagnosis. [file Table_5.docx]

**Supplementary Table 5**. Comparison of general clinical information and sequencing data in patients with a positive and negative molecular diagnosis.

| **WES** | | | | |
| --- | --- | --- | --- | --- |
|  | **Negative** | **Positive** | **Z/Chi-square /t** | **P** |
| Sex (male) | 22(71.0) | 11(47.8) | 2.975 | 0.085 |
| Age (month) | 19.00(8.00-36.00) | 15.00(8.00-21.00) | -1.068 | 0.286 |
| Average depth (X) | 110.22±23.82 | 107.53±26.65 | 0.391 | 0.693 |
| Coverage (%) | 99.87±0.10 | 99.81±0.15 | 1.760 | 0.084 |
| Data size (M) | 8733.84±1607.51 | 8951.98±1897.06 | 0.456 | 0.650 |
| Q20 | 0.97±0.01 | 0.97±0.02 | -0.329 | 0.743 |
| Q30 | 0.93±0.02 | 0.93±0.03 | -0.031 | 0.975 |
| **CNVseq** | | | | |
|  | **Negative** | **Positive** | **Z/Chi-square /t** | **P** |
| Sex (male) | 28(60.9) | 5(62.5) | 0.000 | 1.000 |
| Age (month) | 15.00(7.75-25.25) | 18.50(9.50-32.00) | -0.853 | 0.408 |
| Reads number of CNVseq | 19380511.57±4469695.02 | 20172072.25±1859889.20 | -0.490 | 0.626 |
| Data size (M) | 2796.76±670.09 | 2904.00±254.57 | -0.444 | 0.659 |
| Q20 | 0.97±0.01 | 0.97±0.02 | 0.852 | 0.398 |
| Q30 | 0.93±0.02 | 0.92±0.03 | 0.268 | 0.790 |
